# Relapsing cerebral atypical teratoid/rhabdoid tumor after trimodality therapy: A case report

**DOI:** 10.1097/MD.0000000000027986

**Published:** 2021-11-24

**Authors:** Linlin Meng, Linlin Wang, Guangrui Shao

**Affiliations:** Department of Radiology, The Second Hospital, Cheeloo College of Medicine, Shandong University, Jinan, Shandong, China.

**Keywords:** adolescent, atypical teratoid/rhabdoid tumor, MRI, recurrence

## Abstract

**Introduction:**

Atypical teratoid rhabdoid tumor (AT/RT) is a high-grade embryonal malignant neoplasm of the central nervous system. It is rare and most often diagnosed in children <4 years of age. The biological manifestations of AT/RTs are highly malignant and have a very poor prognosis. Here, we present the case of a 16-year-old boy with AT/RT in the right parietal lobe and with a dismal outcome.

**Patient concerns:**

A 16-year-old male boy presented with a headache after waking up for 1 year without obvious cause. The pain was persistent and dull, mainly in the right orbital, and was slightly relieved after pressing the orbital. Occasionally, nausea and vomiting occurred, and the vomiting was gastric contents. Examination and head computed tomography performed at a local hospital revealed a space-occupying lesion in the right parietal lobe. The patient was then transferred to our hospital for further diagnosis and treatment.

**Diagnosis:**

The patient underwent craniotomy and gross total excision of the tumor. Further histologic examination of the tumor was identified (space-occupying lesion in the right parietal lobe) AT/RT, World Health Organization grade IV.

**Interventions:**

The patient was transferred to the oncology department for radiotherapy and chemotherapy after surgery recovery.

**Outcomes:**

The patient did not comply with the advice for adjuvant chemotherapy regularly and the tumor recurred rapidly. Finally, the patient died after 18 months after the definitive surgery.

**Conclusion:**

In conclusion, in the presence of a tumor with peripheral cystic components or hemorrhage in young children, a diagnosis of AT/RT must always be considered. Patients must follow the doctor's advice for active treatment. All relevant data are within the paper and its Supporting Information files.

## 1 Introduction

Atypical teratoid rhabdoid tumor (AT/RT) is an aggressive malignancy that occurs predominantly in infants. It can occur in any part of the nervous system, and approximately 50% of cases occur in the posterior fossa.^[1–3]^ It was first described by Rorke et al^[4]^ and was defined as a grade IV tumor according to the 2016 World Health Organization classification of central nervous system tumors.^[5]^ AT/RTs are associated with the deletion of chromosome 22q and inactivation of INtegrase Interactor 1 tumor suppressor gene located on chromosome 22q11.2.^[6,7]^ Most AT/RTs are predominantly characterized by rhabdoid cells, with additional epithelial, neuroepithelial, and mesenchymal constituents.^[8,9]^ The clinical presentations and neuroimaging features of patients with AT/RT often lack specificity.^[10]^ The aim of this case report was to focus on the imaging findings and poor prognosis of AT/RT in adolescent patients.

## 2 Case presentation

### 2.1 Patient history

A 16-year-old boy presented with a headache after waking up for 1 year without obvious cause. The pain was persistent and dull, mainly in the right orbital, and was slightly relieved after pressing the orbital. Occasionally, nausea and vomiting occurred, and the vomiting was gastric contents. He was admitted to a community hospital, where he was considered to have “cervical spondylosis” and was administered local massage treatment, but the condition did not improve significantly. Examination and head computed tomography performed at a local hospital revealed a space-occupying lesion in the right parietal lobe. The specific diagnosis and treatment processes at the local hospital are unknown. The patient was then transferred to our hospital for further diagnosis and treatment. This study was approved by the Institutional Review Board of The Second Hospital, Cheeloo College of Medicine, Shandong University.

### 2.2 Neurological examination findings

Physical examination revealed no sensory or motor disorders. His hearing response was normal. Pupillary reflex and eye movements were normal. The patient denied having a personal or family history of cancer, specifically brain tumors. Cerebrospinal fluid was negative for alpha-fetoprotein and placental alkaline phosphatase.

### 2.3 Neuroimaging findings

**Figure 1:**
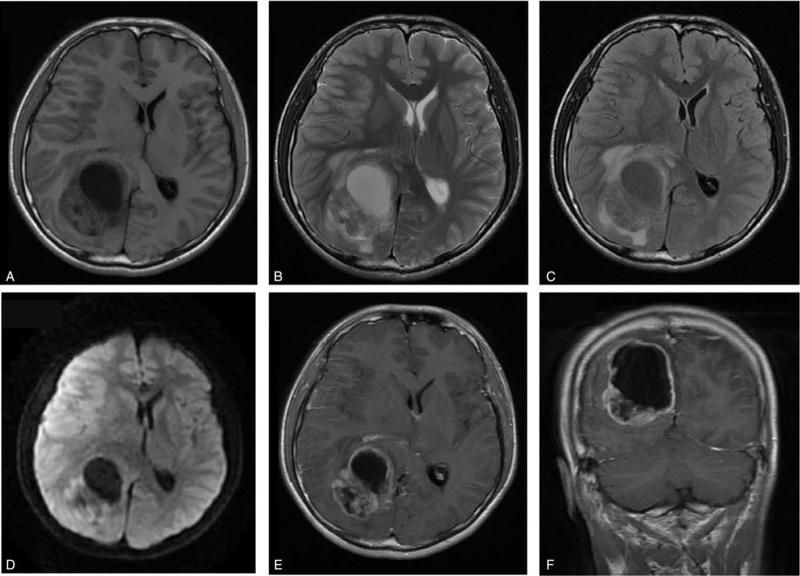
Preoperative magnetic resonance imaging. A, Axial T1-weighted images demonstrated a well-circumscribed solid-cystic mass measuring 3 cm in the right parietal lobe. The mass displayed hypointensity for the most part with a cystic component showing very low intensity. Prominent peritumoral edema and midline shift were seen. B, On axial T2-weighted images, the mass showed heterogeneous high intermediate intensity. C, On axial T2flair-weighted images, the mass showed heterogeneous low intermediate intensity. D, On diffusion-weighted images, the mass showed low and slightly high intensity. E, F, Axial and coronal T1-weighted images with administration of the contrast medium demonstrated a well-demarcated lobular mass with heterogeneous enhancement. A cystic component with no enhancement was seen in the mass.

Magnetic resonance imaging (MRI) of the brain demonstrated a 3.0 cm × 2.0 cm × 1.8 cm well-circumscribed solid-cystic mass located in the right parietal lobe, with hypointense and patchy hyperintense on T1-weighted images, mixed hyperintense on T2-weighted images, iso- to hypointense on FLAIR images, and hypointense on diffusion-weighted images. There was edema of the white matter around the tumor. Contrast-enhanced imaging of the lesions showed strong enhancements of the solid part and cyst wall. However, the cystic components were not enhanced. The posterior horn of the right lateral ventricle and sulci around the lesion were deformed due to tumor compression. The midline was shifted to the left side (Fig. 1).

### 2.4 Diagnosis and treatment

The patient underwent craniotomy and gross total excision of the tumor. The tumor was grayish–white, soft, friable, mildly vascular, with areas of multifocal hemorrhage/necrosis. Further histologic examination of the tumor was identified (space-occupying lesion in the right parietal lobe) AT/RT, World Health Organization grade IV.

**Figure 2:**
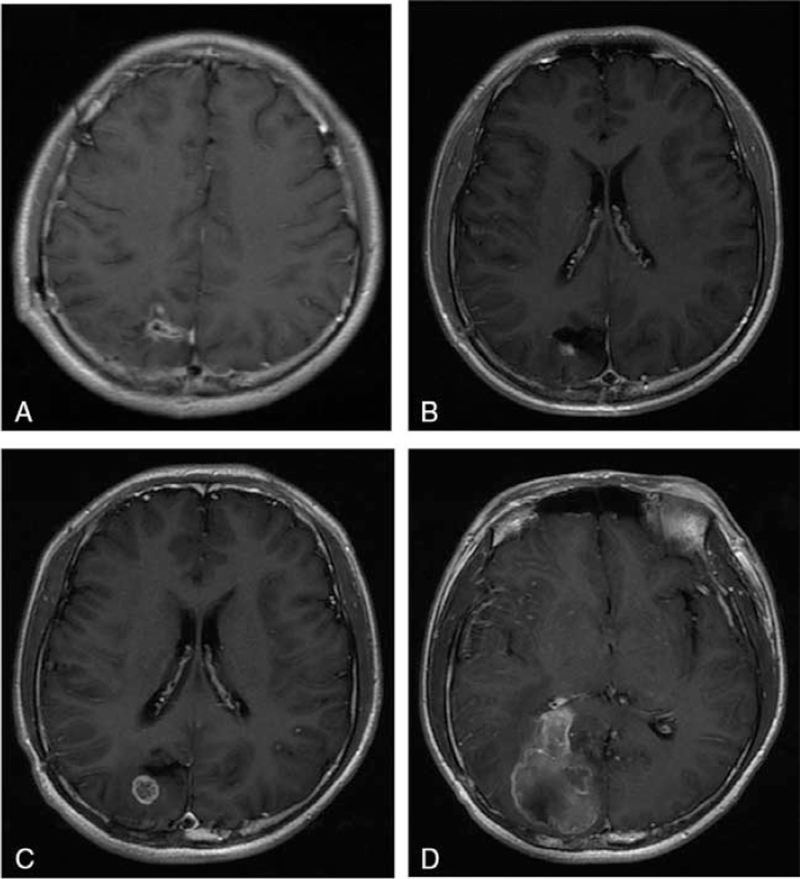
Postoperative imaging. A, Brain MRI obtained at first time after operative demonstrating gross total resection and consistent with favorable radiotherapy treatment response. B, Brain MRI showed nodular enhancement could be seen on the enhanced imaging. C, MR scan revealed increased lesion range, with evidence of tumor recurrence. D, Brain MRI obtained demonstrating enlargement of the tumor, with unclear boundaries and significantly heterogeneous enhanced. MRI = magnetic resonance imaging.

The patient had a stable postoperative course and was discharged after a week with a scheduled follow-up appointment. About 8 months after the operation, the brain MR showed no signs of residual or recurrence of the tumor on January 6, 2019 (Fig. 2A). Subsequently, the patient was transferred to the oncology department for radiotherapy and chemotherapy after surgery recovery. The patient tolerated the adjuvant radiotherapy and chemotherapy regimen well and without complications. On March 18, 2019, the patient was admitted to the neurosurgery department again, and cerebral MRI showed that part of right parietal lobe was missing after surgery, with patch long T1 and long T2 signal areas, and the surrounding signals were not homogeneous. A small ring enhancement was observed on enhanced imaging (Fig. 2B). On April 15, 2019, the patient was admitted to the neurosurgery department. The patient relatives refused chemotherapy and were not compliant with the advice of adjuvant therapy. The patient experienced occasional dizziness and no other apparent discomfort on September 15, 2019. Brain MRI showed a contrast-enhancing mass composed of both cystic and solid areas in the right occipital lobe, with unclear boundaries (Fig. 2C). The lesion showed significant heterogeneous enhancement with obvious marginal enhancement. Tumor recurrence was considered, and the patient was recommended to be transferred to oncology department. Doctors explained the condition to the patient's family repeatedly, and the patient's family said that they informed consent and would make a decision after consultation. One week before March 23, 2020, he experienced headache, accompanied by vomiting, no blurred objects and no dizziness, which made him readmitted to the our hospital. MRI revealed enlargement of the tumor, 4.2 × 2.3 × 2.5 cm in size (Fig. 2D), showed evidence of progress of tumor. Finally, the patient died after 18 months after the definitive surgery. Table 1 shows the patient's medical history.

**Table 1:** Medical history timeline.

September, 2018	Symptoms of headache, nausea, vomiting, CT at a local hospital revealed a space-occupying lesion in the right parietal lobe
November 26, 2018	Neurosurgery department visit with referral for further treatment
November 29, 2018	Brain MRI obtained demonstrating solid-cystic mass located in the right parietal lobe (Fig. 1)
December 02, 2018	Subparietal craniotomy for mass removal
January 06, 2019	Brain MRI obtained demonstrating gross total resection and consistent with favorable radiotherapy treatment response (Fig. 2A)
January 07, 2019	Postoperative adjuvant radiotherapy begins
January 14, 2019	Postoperative adjuvant chemotherapy begins
January 25, 2019	Discharged from hospital
March 17, 2019	Postoperative visit—symptomatic improvement and wound healing well
March 18, 2019	Follow-up brain MRI obtained and with evidence of recurrence (Fig. 2B)
March 19, 2019	postoperative adjuvant chemotherapy begin
April 15, 2019	Postoperative visit, rejected regular chemotherapy
September 15, 2019	Brain MRI obtained demonstrating increased lesion range, indicating the progress of tumor (Fig. 2C)
February 25, 2020	Symptoms of headache, vomiting
March 23, 2020	Brain MRI obtained demonstrating enlargement of the tumor (Fig. 2D)
June 21, 2020	Patient expired

### 2.5 Pathological findings

On pathological sectioning, microscopic examination revealed histological evidence of rhabdoid cells, with abundant eosinophilic cytoplasm, and large and eccentrically placed nuclei. Tumor cell atypia was evident, and mitosis was common.

**Figure 3:**
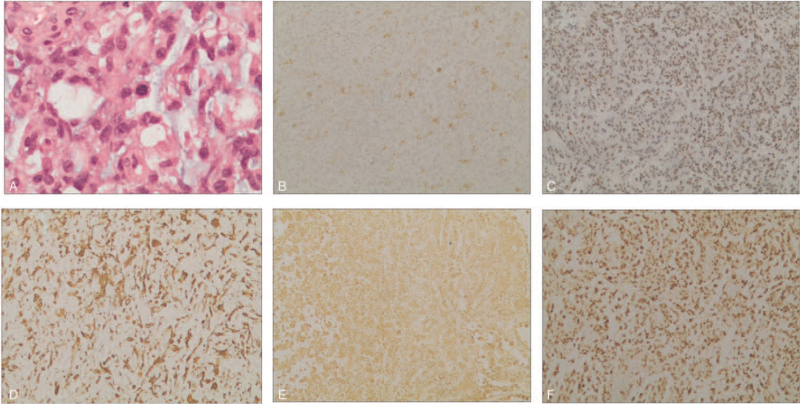
Pathological findings. A, HE staining, Original magnification 400. B–F, Immunohistochemical staining, Original magnification 100. A, HE staining showed that tumor cells had rhabdomyoid cells with epitheloid morphology with prominent nucleoli and high mitotic activity, (B) EMA (+), (C) FLI-1 (+), (D) GFPA (+), (E) Vimentin (+), (F) Olig-2 (+). EMA = epithelial membrane antigen, FLI-1 = friend leukemia virus integration 1, GFAP = glial fibrillary acidic protein, Olig-2 = oligodendrocyte lineage transcription factor 2.

Immunohistochemical analysis of the neoplastic cells revealed positive reactions of antibodies to epithelial membrane antigen, vimentin, glial fibrillary acidic protein, Friend leukemia virus integration 1, oligodendrocyte lineage transcription factor 2, and vimentin (Fig. 3).

## 3 Discussion

AT/RTs are rare and aggressive neoplasms that account for approximately 6% of the central nervous system tumors in pediatric population.^[11]^ Tumor diagnosis of is ultimately dependent on the pathology. The clinical manifestations of AT/RT are related to tumor location and are, therefore, not specific. Because of its malignancy, AT/RTs have a high rate of local relapse and subarachnoid dissemination in the early stages, with 30% to 40% of patients presenting with metastases at the time of detection.^[12]^ Treatment approaches are multimodal, and the widely used treatment is combination of surgical resection with systemic chemotherapy and radiation therapy.^[13]^ High-dose chemotherapy with stem cell rescue may have value for AT/RTs and has increasingly become a popular and mainstay of adjuvant therapy following surgery.^[9,14,15]^ Despite the use of adjuvant chemotherapy and/or radiotherapy, the prognosis is poor, with a mean survival time of less than 1 year.^[9]^ Death from tumor progression occurred very rapidly in children who were not treated at the time of diagnosis. Once progression occurred in the patients being treated, death occurred rapidly. In this report, we describe a case of primary cerebral AT/RT in a young boy. The patient underwent a gross surgical resection of the mass, followed by chemotherapy and radiotherapy with no acute sequelae and partial regression of the previous symptoms. The patient was not compliant with the advice for adjuvant therapy, which led to the tumor recurrence.

Radiological findings of AT/RTs are nonspecific. They were isointense to slightly hyperintense to the gray matter on T1-weighted images and heterogeneous intermediate hyperintense on T2-weighted images, with multiple necrotic foci, cysts at the periphery of the tumor, hemorrhage, and variable contrast enhancement.^[16–20]^ Peritumoral edema is sometimes observed around the tumor. Similar to these characteristics, necrosis, hemorrhage, cystic changes, and peritumoral edema were also observed in our case. A previous study has described an unusual pattern of band-like enhancement zones surrounding the cystic or necrotic areas ^[21]^

Due to its histological and imaging similarities with other neoplastic processes with rhabdoid features, AT/RT is often misdiagnosed. The most common radiological differential diagnosis for AT/RTs in children consists of primitive neuroectodermal tumors or medulloblastomas.^[22–25]^ Clinically, children with AT/RTs have a much worse prognosis than children with primitive neuroectodermal tumors /medulloblastomas, with little or no response to chemotherapy or radiation therapy, and death usually occurs within a year.

## 4 Conclusion

In conclusion, in the presence of a tumor with peripheral cystic components or hemorrhage in young children, a diagnosis of AT/RT must always be considered. Patients must follow the doctor's advice for active treatment.

## Acknowledgment

The authors thank Lulu Zhang for preparing the pathological figure.

## Author contributions

**Resources:** Linlin Wang.

**Writing – original draft:** Linlin Meng.

**Writing – review & editing:** Guangrui Shao.
