# Relapsing cerebral atypical teratoid/rhabdoid tumor after trimodality therapy: A case report: Erratum

**DOI:** 10.1097/MD.0000000000028579

**Published:** 2022-01-14

In the article, “Relapsing cerebral atypical teratoid/rhabdoid tumor after trimodality therapy: A case report”^1^, which appears in Volume 100, Issue 47 of *Medicine*, the caption for Figure 1 appeared incorrectly. The descriptors for panels D-F were mislabeled as E-G. The caption now appears as “Preoperative magnetic resonance imaging. A, Axial T1-weighted images demonstrated a well-circumscribed solid-cystic mass measuring 3 cm in the right parietal lobe. The mass displayed hypointensity for the most part with a cystic component showing very low intensity. Prominent peritumoral edema and midline shift were seen. B, On axial T2-weighted images, the mass showed heterogeneous high intermediate intensity. C, On axial T2flair-weighted images, the mass showed heterogeneous low intermediate intensity. D, On diffusion-weighted images, the mass showed low and slightly high intensity. E,F, Axial and coronal T1-weighted images with administration of the contrast medium demonstrated a well-demarcated lobular mass with heterogeneous enhancement. A cystic component with no enhancement was seen in the mass.”
